# Study on the current status of self-perceived burden and its correlation with caregiver reactions in spinal cord injury patients under the background of medical consortium

**DOI:** 10.3389/fpsyg.2025.1579861

**Published:** 2025-05-26

**Authors:** Jie Jiang, Jingjun Xie, Jianwei Sun, Qi Sun

**Affiliations:** Department of Rehabilitation Medicine, The First People's Hospital of Huzhou, Huzhou, Zhejiang, China

**Keywords:** medical consortium, spinal cord injury, self-perceived burden, caregiver reaction, psychosocial interventions

## Abstract

**Objective:**

The current study tried to investigate the self-perceived burden (SPB) status and its influencing factors in spinal cord injury (SCI) patients under the background of medical consortium, and to analyze the relationship between SPB and caregiver reactions.

**Methods:**

A total of 120 SCI patients treated within the Huzhou medical consortium were selected as study subjects using a convenience sampling method. Data were collected using a general information questionnaire, the SPB Scale, and the Caregiver Reaction Assessment (CRA). Multiple linear regression analysis was used to identify the influencing factors of SPB, and Spearman correlation analysis was used to examine the relationship between SPB and caregiver reactions.

**Results:**

The mean SPB score for SCI patients within the medical consortium was (31.06 ± 9.10). Multiple linear regression analysis revealed that patients’ ability to perform activities of daily living, SCI-related complications, per capita monthly household income, and marital status were independent factors influencing SPB (*p* < 0.05). SPB was positively correlated with the health problems, economic problems, time disruption, and lack of family support dimensions of the caregiver reaction assessment, while it was negatively correlated with the self-esteem dimension (*p* < 0.001).

**Conclusion:**

The SPB of SCI patients is at a moderate level and is correlated with caregiver reactions. Rehabilitation professionals should actively guide caregivers, enhance their emotional regulation abilities, and reduce the SPB of patients.

## 1 Introduction

Spinal cord injury (SCI) is a significantly debilitating condition affecting the central nervous system, characterized by the structural damage of the spinal cord and subsequent loss of functionality (Zipser et al., 2022). This injury can result from external mechanical trauma, such as vehicular accidents or falls from elevated surfaces, as well as from internal pathological factors, including tumor-induced compression or ischemic conditions affecting the spinal cord (Zipser et al., 2022). Clinically, it manifests as dysfunction in movement, sensation, and autonomic nerve functions below the level of injury. The combination of abrupt onset and protracted recovery often necessitates that patients engage in a multifaceted psychological adaptation process (Soendergaard et al., 2019). The global burden of disease associated with spinal cord injury (SCI) has markedly increased over the past 30 years, with prevalence rates escalating from 236 to 1,298 cases per million individuals. Furthermore, annual incidence rates are projected to reach between 250,000 and 500,000 cases, highlighting the considerable public health challenges posed by this condition (Khorasanizadeh et al., 2019). While it is estimated that 60–70% of adult patients are able to gradually adjust to their disabilities, nearly all individuals with SCI experience traumatic grief responses during their recovery process. Additionally, approximately 40% of these patients are at risk for subclinical or clinically significant psychological distress and psychiatric disorders, indicating a psychological crisis that interacts intricately with their physical impairments (Sandalic et al., 2022).

In the context of disease adaptation, self-perceived burden (SPB) emerges as a significant psychological challenge that substantially influences patient prognosis (Cousineau et al., 2003; Ren et al., 2020). SPB is associated with the onset of negative emotional states, including anxiety, depression, and guilt, and it contributes to a detrimental cycle that diminishes quality of life and intensifies social isolation (Gong et al., 2020). Zeng et al. (2022) found that a certain level of SPB can affect patients’ decision-making, lead to treatment refusal, ineffective coping strategies, and diminished survival desire. Therefore, alleviating SPB in SCI patients is particularly important. The concept of caregiver burden was first introduced by Grad and Sainsbury (1966), referring to the costs borne by families and the negative impact on them during the caregiving process. Based on this, caregiver reactions (i.e., the subjective feelings of caregivers during the process of caring for the patient) are also believed to influence the patient’s SPB. Such an influence has been reported in studies involving patients following hip replacement surgery (Xu et al., 2018), those undergoing hemodialysis (Arechabala et al., 2011), and stroke patients (Ren et al., 2020). However, there is still a lack of evidence-based research for the SCI population that needs to be explored in depth.

To address this multidimensional health challenge, the medical alliance model based on hierarchical diagnosis and treatment demonstrates unique integration advantages. This model facilitates vertical collaboration among tertiary hospitals, rehabilitation centers, and community healthcare services, thereby establishing a comprehensive intervention chain that encompasses acute treatment, subacute rehabilitation, and chronic management (Hu et al., 2016). By leveraging the collaborative efforts of multidisciplinary teams—including specialists in neurosurgery, orthopedics, rehabilitation medicine, and psychiatry—tailored and holistic treatment plans can be developed for patients. This approach not only effectively mitigates the burden of specific health conditions but also enhances the responsiveness of caregivers. This collaborative approach offers personalized and comprehensive treatment plans, which can help improve both the patients’ SPB and the caregivers’ responses. Based on the literature review and theoretical analysis, this study proposes that secondary SPB in patients may be significantly associated with caregiver health literacy, socioeconomic status, and family support systems. Therefore, this study focuses on relevant explorations, aiming to systematically analyze the epidemiological characteristics of SPB and its influencing factors in SCI patients, exploring the interaction between SPB and caregiver responses to inform a comprehensive mind–body intervention strategy based on empirical evidence.

## 2 Materials and methods

### 2.1 Study subjects

A total of 120 patients who underwent treatment for SCI in a medical consortium in Huzhou from January 2023 to December 2024 were selected as study subjects. All participants underwent a standardized rehabilitation program. Inclusion criteria: (1) Patients receiving SCI rehabilitation treatment, with their primary caregivers being family members (e.g., parents, children, siblings) responsible for caregiving; (2) Age ≥ 18 years; (3) No history of mental illness, and no communication barriers in speech; (4) The level of spinal cord neurologic injury is consistent with the International Standards for Neurologic Classification of Spinal Cord Injury (ISNCSCI) Grade D or worse as promulgated by the American Spinal Cord Injury Association (ASIA) in 2019 (Rupp et al., 2021); (5) Informed consent for participation in the study. Exclusion criteria: (1) Cognitive impairment, sensory or auditory disabilities, or comorbid traumatic brain injury disorders; (2) History of severe cardiovascular diseases, malignancies, or other serious conditions; (3) Other neurological diseases unrelated to SCI, such as stroke, Parkinson’s disease, or Guillain-Barré syndrome; (4) Patients in critical condition who were unable to participate in the survey; (5) Caregivers who were non-family members receiving remuneration for caregiving. This study was approved by Huzhou First People’s Hospital’s ethics committee, with the ethics approval number 2022GZB05.

### 2.2 Methods

#### 2.2.1 Survey tools

(1) General Information Questionnaire: this included demographic information such as patient gender, age, marital status, educational level, employment status, disease duration, household per capita monthly income, primary disease, medical expense coverage, ability to perform daily activities, presence of complications, caregiver identity and caregiver health status.

(2) SPB Scale: the SPB scale was developed by Cousineau et al. (2003), with a Cronbach’s *α* coefficient of 0.874. The scale includes three dimensions: physical, emotional, and economic burden, with a total score range of 10–50 points. Based on SPBS scores, the burden levels are classified as: no significant burden (<20), mild burden (20–29), moderate burden (30–39), and severe burden (≥40).

(3) Caregiver Reaction Assessment (CRA) Scale (Kew and Osborne, 2024): this scale includes 24 items across five dimensions: self-esteem, health problems, economic problems, time disruption, and lack of family support. The self-esteem dimension includes six statements, such as “This caregiving role makes me feel valuable” and “I take pride in my ability to perform this caregiving task.” These statements highlight the positive emotions caregivers experience, including a sense of self-worth, acknowledgment of their skills, and feelings of personal growth. The health problems dimension contains seven items, like “Caregiving leaves me physically exhausted” and “My health suffers because of caregiving.” These items address both physical fatigue and pain, as well as the psychological stress, anxiety, and depression that can arise from caregiving. The economic problems dimension features three statements, such as “I worry about the costs associated with caregiving” and “I have lost income due to caregiving,” which illustrate the financial strain and resource limitations caregivers encounter. The time disruption dimension includes four statements, such as “I feel that caregiving consumes my time” and “I struggle to find time for my own activities.” This indicates that caregiving significantly occupies the caregiver’s time, making it challenging to manage personal and leisure activities, thus disrupting their daily routine. Lastly, the lack of family support dimension consists of four statements, including “I receive little support from my family” and “I feel my family does not understand the challenges I face.” These reflect the caregiver’s experience of insufficient support, assistance, and understanding from family members during the caregiving process. Each item is rated on a 5-point scale, ranging from “strongly disagree” (1 point) to “strongly agree” (5 points). The self-esteem dimension represents a positive feeling, where higher scores indicate less pressure, while higher scores in the remaining four dimensions indicate greater caregiver stress. The scale has a reliability and validity of 0.9 (Zheng et al., 2008).

#### 2.2.2 Survey method

The research team provided standardized guidance in a uniform language to explain the instructions for completing the questionnaires. Paper-based questionnaires were distributed to the patients, who filled them out on-site. For patients who were unable to complete the questionnaires independently due to reasons such as language barriers, cultural factors, or age, the researchers assisted them in filling out the forms. A total of 120 questionnaires were distributed among patients and caregivers respectively, and 120 valid questionnaires were successfully collected in this population with a 100% response rate.

### 2.3 Statistical analysis

Statistical analysis was performed using SPSS 23.0 software. For normally distributed data, the mean ± standard deviation ( $\bar{x}$ ± *s*) was used for presentation; for data not following a normal distribution, the median (P25, P75) was reported. Categorical data were expressed as frequencies and percentages. Between-group comparisons were conducted using the rank-sum test and Chi-square test. Multivariate linear regression analysis was employed for multivariable analysis, and correlation analysis was performed using Spearman’s rank correlation. A *p*-value of <0.05 was considered statistically significant.

## 3 Results

### 3.1 Status of SPB and caregiver reaction scores in patients

**Figure 1:**
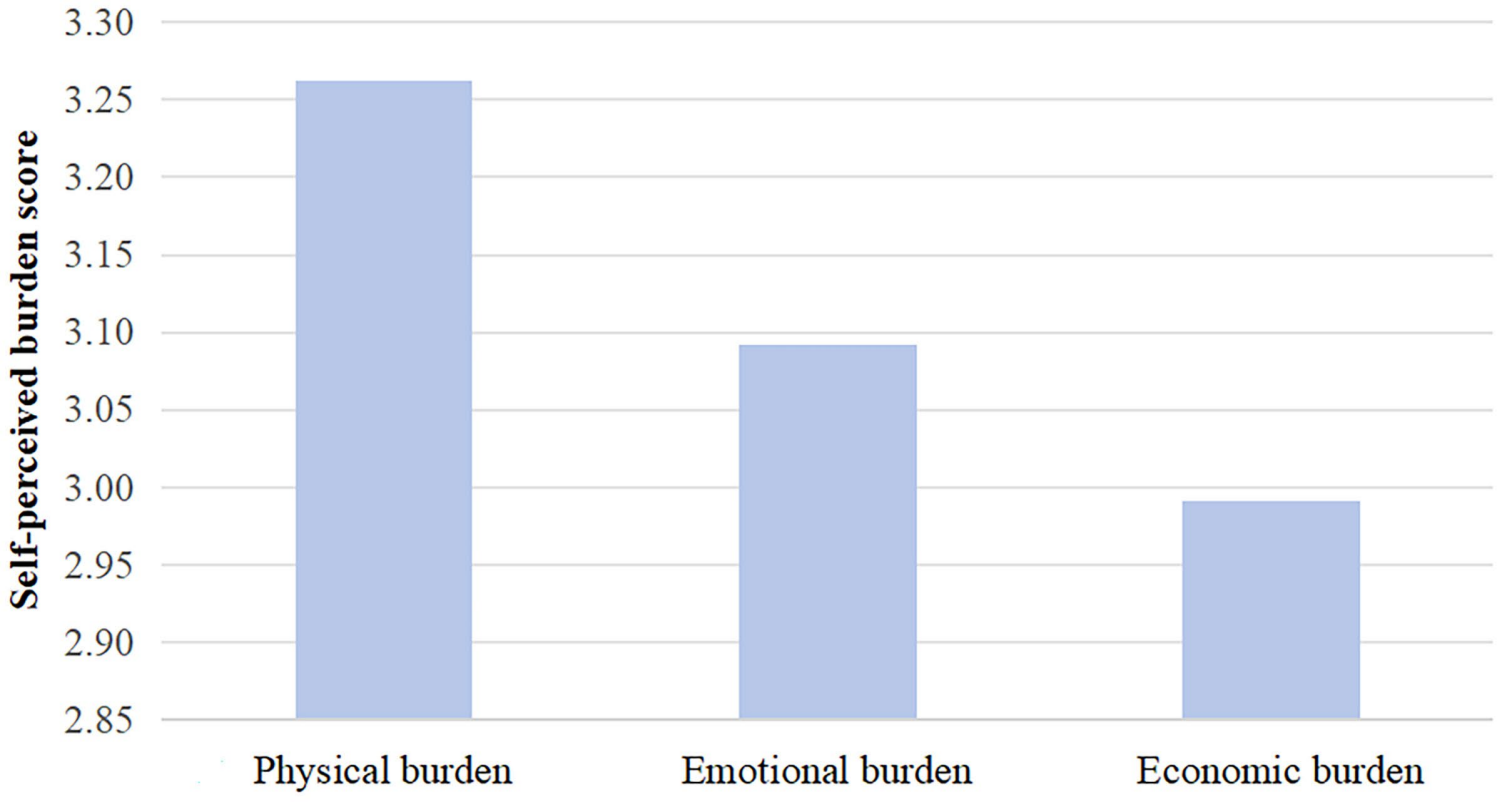
Mean score for self-perceived burden dimension.

**Figure 2:**
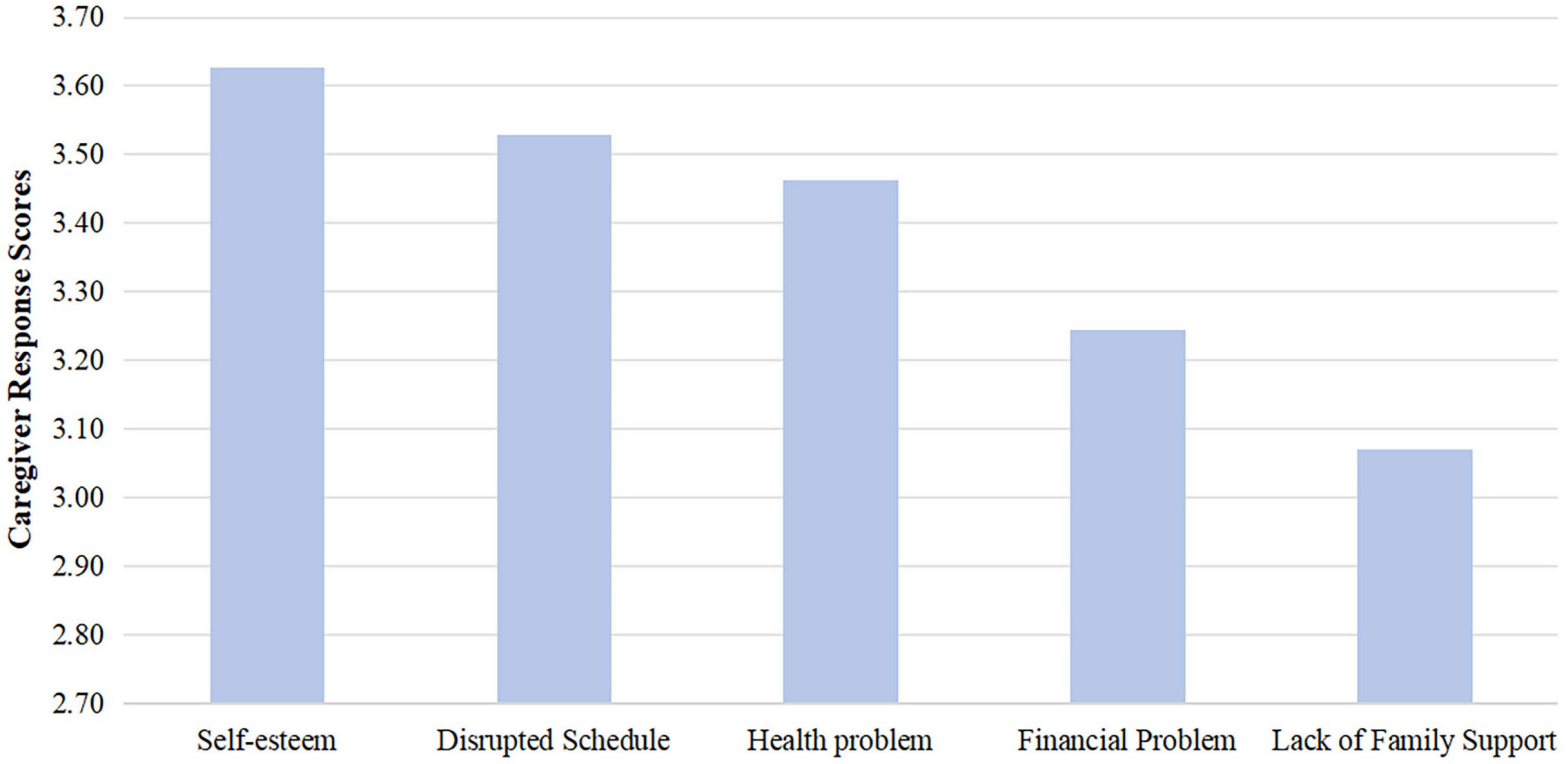
Caregiver response dimension mean score.

A total of 120 SCI patients were included in this study, consisting of 75 males (62.5%) and 45 females (37.5%), with a mean age of (60.38 ± 10.09) years. The total SPB score for the 120 SCI patients was (31.06 ± 9.10), with 7 patients reporting no burden, 45 patients with mild burden, 32 patients with moderate burden, and 36 patients with severe burden. The dimensions with the highest to lowest mean scores were physical burden, emotional burden, and economic burden. In terms of caregiver reactions, the dimensions with the highest to lowest mean scores were self-esteem, time disruption, health problems, economic issues, and family support. The mean scores of patients’ SPB and caregiver reactions for each dimension are shown in Figures 1, 2.

### 3.2 Analysis of factors affecting SPB in patients

The results of the univariate analysis revealed that educational level, marital status, household monthly income, SCI-related complications, payment method, ability to perform daily activities, and differences in payment methods had statistically significant effects on SPB (*p* < 0.05). Using the total SPB score as the dependent variable, and the variables with significant findings from the univariate analysis as independent variables, a multiple linear regression analysis was conducted. The results showed that the ability to perform daily activities, SCI-related complications, household monthly income, and marital status were independent factors affecting SPB in patients (*p* < 0.05) (Table 1).

**Table 1:** Analysis of factors affecting SPB in patients.

Items	Category	Sample size	SPB score Median (P25, P75)	Univariate analysis		Multivariate analysis			
				Statistical value	*P*	*β*	S.E	95%*CI*	*P*
Gender	Male	75	30.00 (22.50 ~ 40.00)	1.551	0.124				
	Female	45	34.00 (24.00 ~ 42.00)						
Age	<60 years	54	30.50 (24.00 ~ 40.00)	0.465	0.643				
	≥60 years	66	33.00 (23.00 ~ 41.00)						
Education level	Primary school or below	95	30.00 (22.00 ~ 35.50)	2.306	0.023	1.185	0.690	0.123(−0.182 ~ 2.553)	0.089
	Middle school	5	42.00 (41.00 ~ 42.00)						
	High school or vocational school	11	41.00 (35.00 ~ 43.00)						
	University or above	9	30.00 (28.00 ~ 41.00)						
Marital status	Married	59	34.00 (25.50 ~ 41.00)	−2.42	0.017	−1.746	0.588	−0.217(−2.911 ~ −0.58)	0.004
	Unmarried	19	31.00 (26.00 ~ 35.00)						
	Divorced	25	27.00 (21.00 ~ 34.00)						
	Widowed	17	28.00 (22.00 ~ 34.00)						
Monthly household income	<3,000 yuan/month	25	40.00 (32.00 ~ 42.00)	−3.127	0.002	−1.865	0.814	−0.168(−3.477 ~ −0.253)	0.024
	3,000 ~ 5,000 yuan/month	15	30.00 (22.50 ~ 34.50)						
	>5,000 yuan/month	80	29.50 (22.00 ~ 38.00)						
Disease duration	<1 year	46	35.00 (24.00 ~ 42.00)	−1.252	0.213				
	1 ~ 3 years	26	32.50 (22.00 ~ 35.00)						
	>3 years	48	30.00 (24.00 ~ 34.00)						
Caregiver’s identity	Spouse	62	31.00 (23.00 ~ 37.00)	0.211	0.833				
	Children	41	33.00 (24.00 ~ 40.00)						
	Parents	12	28.50 (22.00 ~ 42.50)						
	Others	5	30.00 (26.00 ~ 42.00)						
Caregiver’s health	Good	105	31.00 (24.00 ~ 41.00)	−0.845	0.400				
	Poor	15	28.00 (22.00 ~ 34.00)						
Primary disease	Trauma	89	32.00 (24.00 ~ 41.00)	−1.306	0.194				
	Internal causes	31	27.00 (23.50 ~ 34.00)						
Complications of spinal cord injury	None	79	28.00 (22.00 ~ 33.00)	4.973	<0.001	3.308	1.409	0.173(0.517 ~ 6.100)	0.021
	Present	41	40.00 (34.00 ~ 42.00)						
Payment method	Provincial/city medical insurance	85	29.00 (22.00 ~ 40.00)	2.127	0.036	1.836	0.937	0.131(−0.02 ~ 3.692)	0.052
	Rural medical insurance	24	31.50 (26.00 ~ 35.00)						
	Out-of-pocket	11	41.00 (35.00 ~ 42.00)						
Self-care ability	Complete	46	24.50 (21.00 ~ 30.00)	8.539	<0.001	5.496	0.868	0.463(3.777 ~ 7.216)	<0.001
	Partial	47	32.00 (24.50 ~ 41.00)						
	None	27	41.00 (40.00 ~ 42.50)						
Occupation status	Employed	29	28.00 (22.00 ~ 34.00)	1.839	0.068				
	Not employed	91	33.00 (24.00 ~ 41.00)						

### 3.3 Correlation between patients’ SPB and caregiver reactions

The results indicated that patients’ SPB was positively correlated with the dimensions of caregiver health problems, economic issues, time disruption, and lack of family support, while it was negatively correlated with the self-esteem dimension (*p* < 0.05) (Table 2).

**Table 2:** Correlation between patients’ SPB and caregiver reactions.

Items	*R* value	*P*
Self-esteem dimension	−0.279	0.002
Time schedule disruption dimension	0.476	<0.001
Health dimension	0.528	<0.001
Economic issues dimension	0.344	<0.001
Lack of family support dimension	0.375	<0.001

## 4 Discussion

### 4.1 Analysis of the current status of SPB

In this study, the mean SPB score for the 120 patients was (31.06 ± 9.10), indicating a moderate level of SPB, which is consistent with the findings of Zeng et al. (2022). Among the 120 patients, 113 had an SPB score ≥ 20, indicating varying degrees of SPB. The mean scores for each dimension were highest for physical burden (3.26 ± 1.23), followed by emotional burden (3.09 ± 1.17) and economic burden (2.99 ± 1.04), suggesting that physical burden is the most significant aspect of SPB in SCI patients, which aligns with the study by Du et al. (2017). Additionally, SCI often leads to lifelong disability and requires long-term rehabilitation, resulting in significant psychological and economic pressures for patients. As a result, patients may experience feelings of hopelessness and a desire to give up. At the same time, they become highly dependent on family members for financial, emotional, and caregiving support, leading to a sense of special burden and guilt.

### 4.2 Impact of demographic and disease factors on SPB

The results of this study revealed that patients’ ability to perform daily activities, marital status, SCI-related complications, and household monthly income were important factors influencing SPB, which is consistent with the results of similar research (Simmons, 2007; Du et al., 2017). When patients have low self-care abilities and are almost completely dependent on caregivers, this leads to caregiver fatigue and exhaustion. On the other hand, patients may experience a loss of self-worth and diminished self-esteem, becoming more likely to adopt a negative attitude when facing the disease, which intensifies feelings of guilt toward others. Furthermore, compared to married patients, single patients often face prolonged periods of loneliness and lack emotional support, leading to higher SPB scores. Additionally, SCI patients in the recovery phase are prone to complications such as unstable blood pressure, headaches, nausea, vomiting, urinary incontinence, or urinary retention, which not only threaten their physical health but also increase discomfort during treatment, leading to concerns about the effectiveness of treatment (Conti et al., 2019; Faronbi and Olaogun, 2017). On the other hand, due to the impact of the disease, patients often experience a reduction in work capacity or may lose their jobs, leading to a significant drop in income (Du et al., 2017). Moreover, the increased medical expenses during the recovery phase further burden the family’s finances, resulting in a more noticeable SPB (Gordon et al., 2023; Oni-Orisan et al., 2016). Therefore, for recovering patients, healthcare providers should encourage patients to engage in activities they are capable of performing and advise families against over-caring for the patients. At the same time, personalized rehabilitation programs should be selected, with close monitoring of patients’ emotions and psychological symptoms, aiming to reduce complications and provide targeted psychological interventions to encourage patients to reintegrate into society, thereby further reducing their SPB.

### 4.3 Analysis of the current status of caregiver reactions

This study showed that the self-esteem dimension of caregiver reactions (3.63 ± 1.24) had the highest score, which is consistent with the findings of Wang et al. (2023). This suggests that caregivers experience positive feelings during the caregiving process. Influenced by traditional Chinese culture, family members feel a sense of responsibility and obligation to care for patients, which not only provides practical and emotional support to the patient but also affirms their self-worth and personal value. In the negative dimensions, the most prominent issue was time disruption (3.53 ± 1.58), followed by health problems (3.46 ± 1.30). The reason for this is that during the recovery phase of spinal cord injury, caregivers must spend a considerable amount of time accompanying the patient, which leads to a reduction in their own personal time and stagnation in social life. Additionally, caregivers face more demanding tasks compared to the acute phase, such as adjusting the patient’s diet, monitoring intake and output, and maintaining motor function. These caregiving tasks contribute to caregiver physical and mental exhaustion, which adversely affects their health.

### 4.4 Impact of caregiver reactions on SPB

The study found that the higher the caregiver’s self-esteem score, the lower the patient’s SPB. Conversely, the higher the scores for caregiver health problems, economic issues, time disruption, and lack of family support, the more severe the patient’s SPB. Caregiver reactions, as a subjective experience of the caregiver, have a moderating effect on the patient’s SPB (Xu et al., 2018). Positive reactions from caregivers help alleviate the patient’s SPB. However, SCI patients and their caregivers face sudden changes in treatment approaches, a lack of disease knowledge, concerns about the future of both the patient and the caregiver, and various challenges during the caregiving process. These factors lead to negative reactions, and patients often associate their illness with an increased burden on others, resulting in feelings of guilt and self-blame. Therefore, healthcare providers should enhance the assessment of caregiver reactions, provide targeted support that can be intervened upon, and reduce caregiver stress.

### 4.5 Limitations

This study has several limitations that should be noted. Firstly, for certain patients experiencing motor dysfunction, the completion of the questionnaire necessitates assistance from the researcher. This reliance may introduce information bias, potentially stemming from leading questions or social desirability bias, and there exists a risk of underreporting or response bias, particularly concerning the evaluation of psychological and behavioral issues. Secondly, the demographic uniformity of the family caregiver cohort, which predominantly consists of middle-aged and elderly female spouses or parents, may influence their caregiving experiences through latent variables such as gender role expectations and intergenerational communication dynamics. This homogeneity may restrict the applicability of the findings across diverse cultural contexts. Additionally, the inherent limitations associated with causal inference in cross-sectional study designs may hinder the establishment of a temporal relationship between the self-reported behaviors (SPB) and the variables of interest, as well as the complete exclusion of unmeasured confounding factors. Consequently, the validity of the study’s results within the broader population is compromised, thereby diminishing their generalizability. Therefore, future research should prioritize multicenter prospective cohort studies to systematically investigate the dynamic evolutionary trajectory of SPB in patients with spinal cord injury through longitudinal follow-up methodologies.

## 5 Conclusion

This research examined how spinal cord injury patients perceive their own burden and how this relates to the responses of their caregivers within a medical consortium. The study revealed that the psychological burden experienced by patients is significantly linked to the severity of their injuries, the level of social support they receive, and the emotional well-being of their caregivers. Based on these results, it is suggested that a standardized psychological assessment process be implemented in clinical settings. Additionally, the psychological well-being of patients and the reactions of caregivers should be incorporated into routine monitoring systems. A comprehensive intervention model should be developed that combines psychological support, enhanced nursing capabilities, and social care by utilizing various medical resources. These insights offer a theoretical foundation for enhancing the existing rehabilitation service system and suggest a more holistic health management approach for individuals with spinal cord injuries.

## Data Availability

The raw data supporting the conclusions of this article will be made available by the authors, without undue reservation.
